# Drought Tolerance Assessment of Okra (*Abelmoschus esculentus* [L.] Moench) Accessions Based on Leaf Gas Exchange and Chlorophyll Fluorescence

**DOI:** 10.3390/life13030682

**Published:** 2023-03-02

**Authors:** Sonto Silindile Mkhabela, Hussein Shimelis, Abe Shegro Gerrano, Jacob Mashilo

**Affiliations:** 1 Discipline of Crop Science, School of Agricultural, Earth and Environmental Sciences, University of KwaZulu-Natal, Pietermaritzburg 3209, South Africa; 2 Agricultural Research Council—Vegetable, Industrial and Medicinal Plants Private Bag X293, Pretoria 0001, South Africa; 3 African Centre for Crop Improvement (ACCI), University of KwaZulu-Natal, Pietermaritzburg 3209, South Africa

**Keywords:** abiotic stress, chlorophyll fluorescence, drought tolerance, leaf gas exchange, physiological traits

## Abstract

Physiological and complementary phenotypic traits are essential in the selection of drought-adapted crop genotypes. Understanding the physiological response of diverse okra genotypes under drought stress conditions is critical to the selection of drought-tolerant accessions for production or breeding. The objective of this study was to assess the levels of drought tolerance in preliminarily selected okra accessions based on leaf gas exchange and chlorophyll fluorescence to determine best-performing genotypes for drought-tolerance breeding. Twenty-six genetically diverse okra accessions were screened under non-stressed (NS) and drought-stressed (DS) conditions under a controlled glasshouse environment using a 13 × 2 alpha lattice design in three replicates, in two growing seasons. Data were subjected to statistical analyses using various procedures. A significant genotype × water condition interaction effect was recorded for transpiration rate (T), net CO_2_ assimilation (A), intrinsic water use efficiency (WUE*i*), instantaneous water use efficiency (WUE*ins*), minimum fluorescence (*Fo′*), maximum fluorescence (*Fm*′), maximum quantum efficiency of photosystem II photochemistry (*Fv′/Fm′*), the effective quantum efficiency of *PSII* photochemistry (*ɸPSII)*, photochemical quenching (*qP*), nonphotochemical quenching (*qN*) and relative measure of electron transport to oxygen molecules (ETR/A). The results suggested variable drought tolerance of the studied okra accessions for selection. Seven principal components (PCs) contributing to 82% of the total variation for assessed physiological traits were identified under DS conditions. Leaf gas exchange parameters, T, A and WUE*i*, and chlorophyll fluorescence parameters such as the *ɸPSII*, *Fv′/Fm′*, *qP*, *qN*, ETR and ETR/A had high loading scores and correlated with WUE*i*, the *ɸPSII*, *qP* and ETR under DS conditions. The study found that optimal gas exchange and photoprotection enhance drought adaptation in the assessed okra genotypes and tested water regimes. Using the physiological variables, the study identified drought-tolerant accessions, namely LS05, LS06, LS07 and LS08 based on high A, T, *Fm′*, *Fv′/Fm′* and ETR, and LS10, LS11, LS18 and LS23 based on high *AES*, *Ci*, *Ci/Ca*, *WUEi*, *WUEins*, *ɸPSII* and AES. The selected genotypes are high-yielding (≥5 g/plant) under drought stress conditions and will complement phenotypic data and guide breeding for water-limited agro-ecologies.

## 1. Introduction

Okra (*Abelmoschus esculentus* [L.] Moench), belonging to the Malvaceae family, is an important crop mainly cultivated as fruits, vegetables and seed oil. It is extensively grown in tropic and subtropic regions [1] and arid and semi-arid regions with limited and erratic rainfall conditions [2]. The tender and immature pods of okra are consumed as cooked vegetables [3]. The pods are rich in protein content (25 %) and amino acids, notably lysine and tryptophan [4], fat, fibre, vitamins (A, C and K), vital mineral elements such as calcium, potassium, sodium, magnesium, iron, zinc and manganese [5], and soluble sugars such as sucrose (110.4 g/100 g FW), fructose (34.8 g/100 g FW and glucose (30.9 g/100 g FW [6]. In addition, minor quantities of organic acids, including citric, oxalic and malic acid, are present in the succulent pods [6]. The mature and dry seeds are a vital source of edible oils. The seed oil content ranges from 20–40%, consisting of the following major fatty acids: linoleic, palmitic, oleic, diacylglycerols and triacylglycerols acids [7].

Continental Asia accounts for a total annual okra production of 6 million tons from 592,375 million hectares of cultivated land, whereas Africa is the second major producer, with 3 million tons per annum from approximately 1.9 million ha of cultivated land [8]. Commercial and small-scale farmers produce okra. In sub-Saharan Africa (SSA), the crop is mainly grown in marginal conditions characterised by low and erratic rainfall, with minimal agricultural inputs and production technologies. In SSA, okra is mainly cultivated under rainfed conditions, and these agro-ecologies face moderate to severe droughts during the growing season [9]. Drought stress significantly reduces growth, biomass and yield [10]. Drought alone accounts for yield losses ranging between 30 and 100% in okra, primarily when the stress occurs during the flowering and pod-filling stages [3]. Breeding okra cultivars with drought adaptation is the major objective in improvement programs. Physiological and complementary phenotypic traits are critical in the selection of drought-adapted crop genotypes. 

Phenotyping of plants using gas exchange and chlorophyll fluorescence traits has been reported as a preferred approach for selecting drought-tolerant okra accessions [11]. Some gaseous exchange traits used to assess drought tolerance include photosynthesis rate, stomatal conductance, chlorophyll content and transpiration rate. Further, chlorophyll fluorescence parameters (e.g., minimum fluorescence, maximum fluorescence, effective quantum efficiency of *PSII* photochemistry, photochemical quenching and non-photochemical quenching) have been used in phenotyping for drought tolerance [11,12,13]. Drought stress affects okra growth and productivity, disrupting physiological functions and the photosynthetic rate, resulting in yield losses [11,13,14]. Mkhabela et al. [3] reported that okra yield loss under drought stress could be significantly minimised by breeding drought-tolerant ideotypes with intrinsic water use efficiency. Hence, understanding the physiological response of diverse okra genotypes under drought stress conditions is essential for the selection of drought-tolerant accessions for production or breeding.

There has been limited progress in the breeding of okra for drought tolerance. This could be due to limited accessions identified as good drought and heat tolerance sources and insect pests and disease resistance [15]. Some unique accessions, including Sabz Pari [16], NHAe 47-4 [17], Pusa Sawari, Iraq P, Hala [1] and Xianzhi [18], were identified as useful sources of genes for enhancing drought tolerance under water-limited conditions. Compared to the highest genetic diversity reported in the cultivated okra [19], the identified accessions with tolerance to drought are relatively few. Therefore, there is a need for concerted research and development in okra to develop market-led and improved varieties for water-limited conditions. 

In South Africa, okra is an important but under-researched and under-utilised crop. It is grown under rainfed conditions using local and unimproved accessions with poor adaptation and low yield potential. Genetically unique okra accessions could be sourced from different geographical regions to enhance okra pre-breeding programs [3]. Morphological traits associated with drought tolerance in okra include the number of pods per plant, fresh pod length, number of seeds per pod, hundred seed weight, number of branches per plant, plant height and total pod production [1,19]. Reportedly, a higher number of branches, pod length and number of pods per plant, plant height between 150 and 170 cm and pod weight have a direct influence on pod yield [19]. Drought tolerance assessment of okra accessions using the combination of morphological and physiological traits could increase the efficiency of identifying and selecting drought-tolerant accessions for cultivar development under dry environments. Therefore, this study aimed to assess the levels of drought tolerance in preliminarily selected okra accessions based on leaf gas exchange and chlorophyll fluorescence to determine best-performing genotypes for drought-tolerance breeding.

## 2. Materials and Methods

### 2.1. Plant Materials and Study Site

Twenty-five genetically distinct okra accessions were used for the study. The accessions were sourced from the Agricultural Research Council, Vegetable, Industrial and Medicinal Plants (ARC-VIMP) gene bank, and one local variety was included. The accessions were previously studied for their morphological responses to drought stress under field and glasshouse environments [3]. Detailed information on their geographical origin and drought resistance index are presented in Table 1. The experiment was conducted under glasshouse conditions at the Controlled Environment Facility (CEF) of the University of KwaZulu-Natal during the 2020/2021 growing seasons. The first experiment was conducted from September 2020 to December 2020, and the second from February 2021 to May 2021. The accessions were evaluated under non-stressed (NS) and drought-stressed (DS) conditions in the glasshouse environment. Drought tolerance index was calculated as DTI = (Ys/Yn)/(Ms/Mn), where Ys and Yn are the genotype yields under stress and non-stress, and Ms and Mn are the mean yields of the accessions under stressed and non-stressed conditions, respectively [20].

### 2.2. Experimental Design and Crop Establishment

Five seeds were initially planted in 5 L capacity plastic pots filled with composted pine bark growing media. Later, two plants were established per pot for each genotype. The day and night temperatures in the greenhouse (GH) were 30 °C and 20 °C, respectively, and the relative humidity ranged between 45 and 55% during the study. Inorganic fertilizers consisting of nitrogen (N), phosphorus (P) and potassium (K) were applied at a rate of 120, 30 and 30 kg ha^−1^, based on soil fertility recommendations using urea (46-0-0), phosphorus pentoxide (P_2_O_5_) and potassium oxide (P_2_O), respectively. 

The trials were established using a 13 × 2 alpha lattice design under drought-stressed and non-stressed conditions with three replications. Drought stress was imposed at 50% flowering until physiological maturity by withholding irrigation until the soil water content reached 30% field capacity for plants under DS. The duration of stress was seven days before sampling. Plants under NS conditions were irrigated regularly to maintain soil moisture content at field capacity until physiological maturity. To determine pod yield, plants reached maturity, and pods were harvested sequentially at the soft, most digestible and immature stage. Tensiometers, moisture monitors (Spectrum Technologies, Inc, Chicago, IL, USA), were used to detect soil moisture levels at the root zone. Agronomic performance of the test genotypes was reported in Mkhabela et al. [19]. 

### 2.3. Data Collection

Gas exchange and chlorophyll fluorescence parameters were measured using an LI-6400 XT Portable Photosynthesis system (Licor Bioscience, Inc. Lincoln, NE, USA) integrated with an infrared gas analyser (IGRA) attached to a leaf chamber fluorometer (LCF) (640040B, 2 cm^2^ leaf area, Licor Bioscience, Inc, Lincoln, NE, USA). External leaf CO_2_ concentration (C*_a_*) and artificial saturating photosynthetic active radiation (PAR) were set at 400 µmol mol^−1^ and 1000 µmol m^−2^ s^−1^, respectively. Water flow rate and relative humidity were maintained at 500 µmol and 43%, respectively. The leaf-to-air vapour pressure deficit in the cuvette was maintained at 1.7 kPa to avoid stomatal closure due to low air humidity. Gas exchange and chlorophyll fluorescence measurements were taken on the third half fully formed leaf inside the sensor head. Under both NS and DS conditions, measurements were taken from five plants of each accession.

The following gas exchange parameters were determined: stomatal conductance (gs), net CO_2_ assimilation rate (A), transpiration rate (T), intercellular CO_2_ concentration (C*_i_*) and the ratio of intercellular and ambient CO_2_ (C*_i_*/C*_a_*) concentrations. The ratio of net CO_2_ assimilation rate to intercellular CO_2_ concentration (A/C*_i_*) was computed according to Kitao et al. [21]. The ratio of A and gs was used to compute intrinsic water use efficiency [22] and the ratio of A and T was used to calculate instantaneous water use efficiency) [23]. 

To estimate chlorophyll fluorescence variables, a saturation flash intensity of 1300 µmol m^−2^ s^−1^ was applied. The following parameters were recorded. The minimum (*Fo′*) and maximum fluorescence (*F_m_*′) of light-adapted leaves under natural glasshouse conditions. The steady-state fluorescence (*F_s_*) was also determined in light-adapted photosynthesis. Equation (1) was used to determine the variable fluorescence in light-adapted leaves, while Equation (2) calculated fluorescence changes [24].

*Fv*′ = *Fm*′ *F*_0_′ (1)

Δ*F* = *Fm*′ − *Fs* (2)

Additional chlorophyll fluorescence parameters were estimated according to Evans [25], *Fv*′/*Fm*′, the maximum quantum efficiency of photosystem II photochemistry, the effective quantum efficiency of photosystem II photochemistry (*ɸPSII)*, photochemical quenching (*qP*), non-photochemical quenching (*qN*) and electron transport rate (ETR). The ratio of ETR and A was used to calculate a relative measure of electron transport to oxygen molecules. The alternative electron sink (AES) was calculated as the ratio of photosystem II effective quantum efficiency to net CO_2_ assimilation (A) [26]. Chlorophyll fluorescence was measured using a pulse-amplitude modulated (PAM) fluorometer, which applies a short pulse of light to the sample and measures the resulting fluorescence emitted by the chlorophyll. This measurement provided information on the photosynthetic efficiency and health of the crop. Gas exchange and chlorophyll fluorescence parameters were measured on fully expanded leaves. At the end of the second experiment, yield per plant (YPP) was determined by harvesting fresh pods when 50% of the pods were 3–5 cm long by hand every third day. 

### 2.4. Statistical Analysis

Data were subjected to analysis of variance using Genstat 20th edition (VSN International, Hempstead, UK). The mean data for the two seasons were combined for analysis. Means were separated using Fisher’s protected least significant difference (LSD) test at the 5% significance level. Pearson’s correlation coefficients were calculated using IBM SPSS Statistics 25.0 (SPSS Inc., Chicago, IL, USA) to determine the magnitude of the relationship among physiological traits. Principal component analysis (PCA) based on a correlation matrix was used to identify influential traits under NS and DS conditions using R Studio version 4.0, ggplot2 (R Core Team, 2018). Biplots were built using XLSTAT to determine relationships among the accessions and response variables (physiological traits). Principal component biplot diagrams were used to identify drought-tolerant and drought-susceptible okra accessions using XLSTAT. ClustVis (https://biit.cs.ut.ee/clustvis_large (accessed on 23 November 2022)) was used to visualise the heatmap analysis of physiological traits. 

## 3. Results

### 3.1. Leaf Gas Exchange and Chlorophyll Fluorescence Parameters in Response to Drought 

The effects of genotype, water regime and interaction of genotype × water regime were significantly different for most evaluated traits of leaf gas exchange and chlorophyll fluorescence (Table 2). Drought stress significantly reduced gs, A and A/C*i* among the evaluated accessions (Table 3 and Table 4). Accessions LS02, LS09, LS10, LS17, LS19 and LS26 recorded *gs* values of >0.3 mmol m^−2^ s^−1^ under NS conditions. Under DS, accessions LS04, LS11, LS13 and LS20 recorded *gs* values <0.1 µmol m^−2^ s^−1^. Regarding T, accessions LS03, LS13, LS15, LS19, LS23 and LS24 recorded values ≥ 7.01 mmol H_2_O m^−2^ s^−1^ under NS conditions, while, under DS conditions, genotypes LS01, LS03, LS04, LS08, LS09, LS11, LS12, LS14, LS19 and LS22 recorded T values ≤ 1.00 mmol H_2_O m^−1^ s^−1^. Under NS conditions, A values of ≥ 30 µmol CO_2_ m^−2^ s^−1^ were observed from accessions LS08, LS10 and LS21, while values ≤ 20 µmol CO_2_ m^−2^ s^−1^ were recorded for accessions LS03 and LS06. 

Non-significant (*p* > 0.05) differences were observed among accessions under NS and DS conditions for *Ci*. Okra genotypes LS02 and LS21 exhibited high *A/C_i_* values of 0.23 and 0.28 µmol. mol ^−1^, respectively, under DS conditions compared to other accessions. Significant (*p* < 0.05) differences were observed in C_i/_C_a_ values among accessions under both NS and DS conditions. Intrinsic water use efficiency and instantaneous water use efficiency were increased by drought stress (Table 4). Accessions LS13 and LS20 had the highest *WUE_i_* under drought-stress conditions, with 1438.80 and 1256.10 µmol CO_2_ m^−2^, respectively. The highest WUE*_ins_* values under drought stress were recorded for accessions LS04 (2164.70 µmol·mol^−1^) and LS22 (2161.00 µmolmol^−1^). 

The effect of drought stress on chlorophyll fluorescence parameters among the tested okra accessions are highlighted in Table 2. Chlorophyll fluorescence parameters indicated significant differences for genotype, water regime and genotype x water regime interaction, showing that the evaluated genotypes responded differently under non-stress and drought-stress conditions. Non-significant differences were observed for *Fo′* under non-stress, while significant (*p* < 0.001) differences were recorded under drought-stress conditions (Table 3 and Table 4). Genotypic variability (*p* < 0.001) with respect to *F_m_*′ was observed under non-stress and drought-stress conditions. Drought stress decreased *Fv′/Fm′*, from 0.51 under non-stressed to 0.35 under drought-stressed conditions. The *ɸPSII* varied significantly among the tested genotypes under non-stress and drought-stress conditions. LS07, LS12 and LS19 revealed considerably higher values for *ɸPSII* ≥ 0.40 compared to other genotypes under non-stress conditions. 

Photochemical quenching was significantly reduced from 0.32 to 0.13 by drought stress among the evaluated genotypes, of which LS04, LS12 and LS13 had the highest values of *qP* > 0.40. A variable genotypic response was observed with respect to *qN* under non-stress and drought-stress conditions. The mean for *qN* was higher under drought-stress (1.96) than non-stress conditions (1.39). The *qN* values ranged from 0.68 to 2.80 under non-stress (Table 3) and from 0.66 to 3.75 under drought-stress conditions (Table 4). LS02, LS03 and LS11 revealed *qN* values ≥ 2 under non-stress conditions. Genotypes LS01, LS02, LS10, LS11 and LS18 showed *qN* values ≥ 3 under drought-stress conditions. Non-significant differences were observed for ETR under non-stress conditions, while genotypic variation was observed for ETR under drought-stress conditions. LS08, LS09 and LS17 revealed the highest ETR value of ≥34,541 µmol e^−1^ m^−1^ s^−1^, whereas LS16, LS22 and LS26 showed the lowest ETR ≤ 8071 under DS conditions. Drought stress significantly increased ETR/A (Table 4). The highest ETR/A (≥1542 µmol e µmol-1 CO_2_) was recorded from LS03, LS08, LS09 and LS17 under drought-stress conditions. Drought stress significantly increased AES (154.72) compared to NS (26.98). AES ranged from 12.77 to 61.12 under non-stress and from 64.90 to 562.80 under drought-stress conditions. Yield per plant was significantly reduced, from 7.20 g/plant to 4.31 g/plant, by drought stress among the evaluated genotypes. Accessions LS11, LS19, LS21, LS22 and LS24 had the highest yield (>9 g/plant) under NS conditions, whereas LS05, LS06, LS07, LS08, LS10, LS11, LS18 and LS23 exhibited the highest yield (>5 g/plant) under DS conditions.

### 3.2. Correlation between Leaf Gas Exchange and Chlorophyll Fluorescence Parameters under Non-Stressed and Drought-Stressed Conditions

Pearson correlation coefficients showing relationships among leaf gas exchange and chlorophyll fluorescence parameters among the tested okra accessions under NS and DS conditions are presented in Table 5. Under NS conditions, Ci/Ca was highly and significantly correlated with Ci (*r* = 1, *p* < 0.001), WUE*i* with gs (*r* = −0.75, *p* < 0.001), WUE*ins* with T (r = −0.75, *p* < 0.001) and *ɸPSII* with A/Ci (*r* = 0.61, *p* < 0.001). In addition, *qP* was positively and significantly correlated with A (*r* = 0.55, *p* < 0.05), C*i* (*r* = 0.48, *p* < 0.05) and C*i*/C*a* (*r* = 0.48, *p* < 0.05). ETR was positively and highly significantly correlated with A (*r* = 0.71, *p* < 0.001) and *qP* (*r* = 0.52, *p* < 0.001). Positive and high significant correlation was observed between ERT/A and ETR (*r* = 0.86, *p* < 0.001) and AES and q*P* (*r* = 0.52, *p* < 0.001), while a negative and highly significant association was observed between YPP and A (*r* = −0.69, *p* < 0.001). A significant positive correlation was observed between YPP and ETR/A (r = 0.49, *p* < 0.05), YPP and Ci (*r* = 0.34, *p* < 0.05) and YPP and C*i*/C*a* (r = 0.45, *p* < 0.05), while a negative significant correlation was observed between YPP and qN (*r* = −0.45, *p* < 0.05) under NS conditions.

Under DS conditions, a significant positive correlation was detected between A and gs (*r* = 0.57, *p* < 0.05), while A/C*i* was negatively and highly significantly correlated with C*i* (*r* = −0.61, *p* < 0.001). A highly significant negative association was observed between C*i*/C*a* and A/Ci (*r* = −0.57, *p* < 0.001). WUE*i* was positively and significantly correlated with A (*r* = 0.48, *p* < 0.05), while WUE*ins* was negatively and highly significantly correlated with T (*r* = −0.55, *p* < 0.001). *Fv′/Fm′* was positively correlated with gs (*r* = 0.47, *p* < 0.05). *ɸPSII* was positively and highly significantly correlated with gs (*r* = 0.54, *p* < 0.001), while significantly associated with A (*r* = 0.42, *p* < 0.05) and *Fv′/Fm′* (*r* = 0.46, *p* < 0.05). *qP* was positively correlated with WUEins (*r* = 0.39, *p* < 0.05) and highly significantly correlated with *Fm*′ (r = 0.55, *p* < 0.001). Positive correlations were observed between *qN* and A (*r* = 0.48, *p* < 0.05) and C*i* (*r* = 0.48, *p* < 0.05) and WUE*i* (*r* = 0.43, *p* < 0.05). ETR was positively correlated with gs (*r* = 0.45, *p* < 0.05), A (*r* = 0.45, *p* < 0.05), *Fv′/Fm′* (*r* = 0.53, *p* < 0.001) and *ɸPSII* (r = 0.82, *p* < 0.001). Relative measure of electron transport to oxygen molecules was positively and significantly correlated with WUE*i* (*r* = 0.68, *p* < 0.001) and ETR (*r* = 0.82, *p* < 0.001), while AES was positively correlated with T (*r* = 0.45, *p* < 0.05) and *qP* (*r* = 0.48, *p* < 0.05). YPP was highly positively correlated with C*i* (*r* = 0.66, *p* < 0.001), *Fo′* (*r* = 0.83, *p* < 0.001) and Ci/Ca (*r* = 0.67, *p* < 0.001), while significantly associated with WUE*i* (*r* = 0.48, *p* < 0.05) and Fm′ (*r* = 0.40, *p* < 0.05) and negatively correlated with ETR/A (*r* = −0.60, *p* < 0.001) under DS conditions. 

### 3.3. Principal Component Analysis (PCA) for Leaf Gas Exchange and Chlorophyll Fluorescence Traits

Values of PCA, eigenvalues, percent, and cumulative explained variances are summarised in Table 5. Under NS conditions, seven principal components exhibited eigenvalues > 1 and accounted for 81% of total phenotypic variation. Net CO_2_ assimilation, C_i_, C_i_/C_a_, *qP*, ETR, ETR /A, AES and YPP were positively correlated with PC1, which accounted for 22% of the total variation. PC2 was positively correlated with gs, *Fo′* and *ɸPSII*, whereas WUE_i_ and WU*_Eins_* were negatively correlated with PC2, which accounted for 17% of the total variation. Transpiration rate was negatively correlated with PC3, whereas WUE*_ins_*, *qP* and YPP were positively correlated with PC3, which contributed 11.42% of total variation. A/C*i* positively correlated with PC4 accounted for 10.67% of total variation. PC5 was positively correlated with *Fm*′ and *F_v_′/F_m_′,* contributing 8% of total variation, whereas PC6 was positively correlated with Fm′, contributing 7% of total variation. 

Similarly, under DS conditions, seven PCs with eigenvalues > 1 were detected, which contributed 80% of the total phenotypic variability. Yield per plant was negatively correlated with PC1, whereas *ɸPSII*, ETR and ETR/A were positively correlated with PC1, which accounted for 20% of total variation. Transpiration rate, net CO_2_ assimilation, C_i_, WUE_i_, *F_v_′/F_m_′*, *qN* and YPP were positively associated with PC2, accounting for 18% of the total variation. Stomatal conductance and A/Ci were positively correlated with PC3, whereas Ci and WUE*_ins_* negatively associated with PC3 contributed 14% of the total variation. Net CO_2_ assimilation and *qN* were positively correlated with PC4, whereas ETR/A was negatively correlated with PC4, accounting for 10% of total variation. Instantaneous water use efficiency was positively correlated with PC5, which accounted for 7% of total variation, whereas stomatal conductance and photochemical quenching were positively correlated with PC6, which contributed 6% of total variation. 

**Figure 1:**
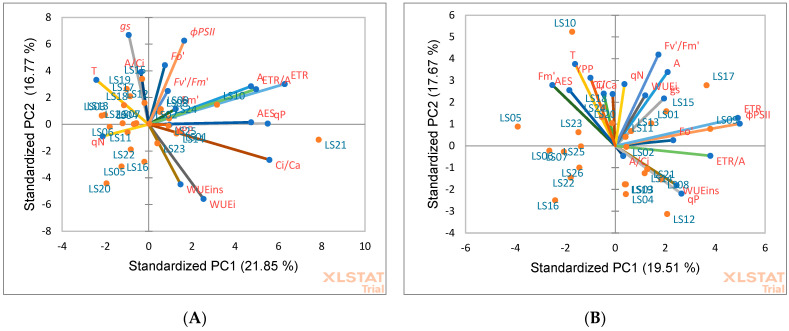
Principal component (PC) biplot of PC1 vs. PC2 depicting the relationships among physiological traits among 26 okra accessions evaluated under non-stressed (**A**) and drought-stressed (**B**) conditions.

**Figure 2:**
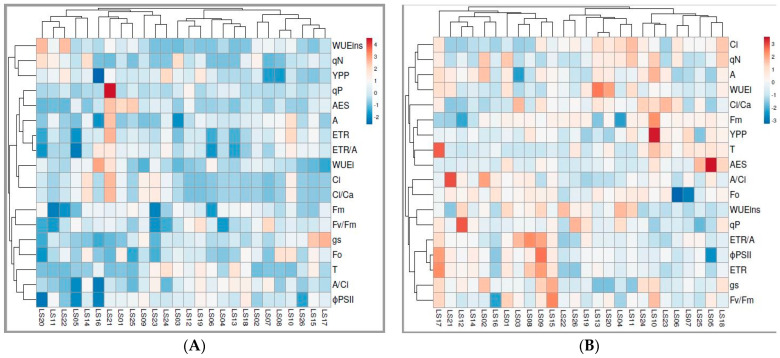
Heatmap showing the relationship among physiological traits among 26 okra accessions evaluated under non-stressed (**A**) and drought-stressed (**B**) conditions.

Principal component biplots based on PCA analysis were used to indicate the relationships among okra accessions for leaf gas exchange and chlorophyll fluorescence parameters under NS (Figure 1A) and DS (Figure 2B) conditions. Traits presented by parallel vectors or those close to each other revealed a strong positive association, and those located nearly opposite (at 180°) showed a highly negative association, while the vectors toward sides expressed a weak relationship. Under NS conditions, accessions LS06, LS11, LS22, LS05 and LS20 were grouped based on high *qN*. Accessions LS19, LS17 and LS18 were grouped together based on high gs, T and A/Ci. LS02, LS10 and LS24 were grouped based on high *ɸPSII*, *Fo′*, *F_v_′/F_m_′*, A, ETR, ETR/A, AES and *qP*. Accessions LS25, LS01, LS23 and LS16 were grouped together based on high C_i_/C_a_, WUE**_i_** and WUE*_ins_*. Under DS conditions, accessions LS10, LS24, LS25, LS05 and LS06 were clustered together based on high *Fm*′, AES, T, C*_i_* and YPP. LS13, LS15, LS17 and LS09 were grouped together based on high C_i_/Ca, *F_v_′/F_m_′*, WUE*_i_* and *ɸPSII*. Accessions LS02, LS19, LS21, LS08 and LS12 were grouped based on high *Fo*′, ETR/A, WUE*_ins_* and *qP*.

### 3.4. Heatmap Analysis for Leaf Gas Exchange and Chlorophyll Fluorescence Traits

A heatmap based on leaf gas exchange and chlorophyll fluorescence traits under NS and DS conditions was constructed using a hierarchical clustering method to discern the relationship of 26 okra accessions based on Jaccard’s coefficient (Figure 2). Under NS (Figure 2A) conditions, physiological traits were grouped into four main clusters. The first cluster consists of two subclusters, dominated by eight accessions, including LS19, LS12, LS06, LS18, LS13, LS07 and LS02, which were grouped based on high negative correlations with WUE*ins*, *qN* and YPP. The second subcluster consisted of accessions LS22, LS11, LS1, LS08, LS20 and LS14, which were negatively correlated with A/Ci and T. LS25, LS01, LS21, LS16, LS24 and LS23 dominated the fourth subcluster under NS conditions and positively correlated with *qP*. Under DS, physiological traits were grouped into three main clusters and six subclusters. The first cluster is dominated by accessions LS19, LS09, LS03, LS15, LS14 and LS17, based on their positive correlations with ETR and ETR/A. LS26, LS22, LS04, LS20, LS13 and LS11 dominated the second cluster under DS conditions, with positive correlations with WUE*i*, WUE*ins*, *qN* and YPP. AES was positively correlated with LS25 and LS05 in the third cluster under DS conditions. 

## 4. Discussion

Okra is one of the most important commercial vegetable crops grown for its fresh fruits and dry seeds. Drought is the major impediment to okra production in dry regions. To adapt to drought stress, plants have undergone many biochemical, molecular, and physiological changes. These changes increase the plants’ tolerance to drought stress. Drought stress influences plant performance by reducing gas exchange and altering chlorophyll fluorescence formation. Gas exchange and chlorophyll fluorescence confer drought tolerance in okra [11,27]. Plants alter gene expression, disrupting the production of photosynthetic pigments and regulating stomatal function to adapt to and tolerate stress conditions [27]. Developing new strategies for maintaining high yield under drought-stress conditions is one of the major challenges in the current crop production system. 

In this study, various physiological drought responses were assessed in okra accessions. Reductions in okra’s stomatal conductance and transpiration rates have been associated with water conservation that allows plants to tolerate drought stress and the loss of physiological functions [9]. Stomatal closure leads to a reduction in CO_2_ assimilation and minimises the rate of water loss through transpiration. This role of drought-induced stomatal closure limits CO_2_ uptake by the leaves and possibly leads to increased susceptibility to photodamage [11]. Similar findings were reported for okra accessions under water shortages [11,27]. These physiological changes increase the plants’ resistance to drought stress, enabling the crop to survive in environments with limited water availability. 

Drought tolerance should be considered as a comprehensive evaluation of carbon assimilation during global climate change challenges [28]. In the current study, okra accessions exhibited a reduction in net CO_2_ assimilation under drought-stressed conditions (Table 4). The decrease in net CO_2_ assimilation during water-stressed conditions might be reversible initially. However, drought in the pod-filling stage might cause irreversible damage to the photosynthetic pathway, thereby affecting carbon assimilation [29]. Further, utilisation of assimilates is relevant in addition to the photosynthetic performance of leaves. The evaluated okra accessions revealed high water use efficiency under drought-stressed conditions (Table 4). Enhancing water use efficiency to sustain okra production under water-limited conditions remains the most important task for water management. Hence, specific responses for enhancing water use efficiency could be achieved with more precise data on crop stress detection [11]. Drought-tolerant accessions exhibited high WUE_i_ and WUE*_ins_* compared to drought-susceptible accessions (Table 2). This indicates that the evaluated accessions use water efficiently, attributed to drought escape mechanisms such as the transpiration rate. Drought-tolerant accessions use water efficiently, maintain tissue water status, reduce water loss and produce stable yield during water shortages [30]. 

Chlorophyll fluorescence is a non-invasive measurement detecting the authenticity of photosystem II [31]. Chlorophyll fluorescence parameters, including photosystem II photochemistry, minimum fluorescence, maximum fluorescence, photochemical quenching and electron transport rate are useful for detecting drought-stress severity, genetic variation and determining damage to *PSII* [32]. *F_v_′/F_m_′* is considered the most important parameter of chlorophyll fluorescence, widely used to evaluate drought-stress response. In this study, a reduced *F_v_′/F_m_′* value was recorded under drought-stress conditions, corroborating with results reported by Ahmed and El-Sayed, [27]. According to Paknejad et al. [33], reduced *F_v_′/F_m_′* under drought-stress conditions indicates the presence of a protective mechanism of light absorption in response to water shortages. Hence, the *F_v_′/F_m_′* parameter can be applied to determine the potential efficiency of *PSII.*

In the present study, drought-tolerant okra accessions showed an efficient photosynthetic affinity compared to sensitive accessions. Photosystem *II* is highly drought tolerant. However, under drought-stress conditions, photosynthetic electron transport through *PSII* is inhibited [24]. The decrease in *PSII* might be due to the photo-protective increase in thermal energy dissipation induced by the excess of absorbed light [34]. However, there are contradictory reports on the direct effect of *PSII* functionality under drought-stress conditions. A study reported that, under mild water stress, *PSII* is not affected [35], while another study reported that, under drought-stress conditions, damage occurs to both photosystem I and photosystem II [36]. The current study found that *PSII* was significantly affected by drought stress. Under drought-stress conditions, the *PSII* thermal energy dissipation was strongly limited due to damage to *PSII* structure and functionality. A decrease in photochemical quenching was observed in the studied okra accessions under drought-stress conditions. Similar results were reported by Ashraf et al. [37] in the study of gas exchange characteristics and water relations in some elite okra cultivars under water-deficit conditions. The decrease in *qP* is attributable to either a decrease in the rate of consumption of reductants and ATP produced from non-cyclic electron transport relative to the rate of excitation of open *PSII* reaction centres or damage to *PSII* reaction centres [24]. 

Positive correlations were observed between non-photochemical quenching and intrinsic water use efficiency under drought-stress conditions, indicating a protective mechanism by the plants against reactive oxygen species that harm antenna pigments and closing reactions in the photosystem. Drought stress also affects the electron transport rate (ETR) and alternative electron sink (AES) [38]. An increase in alternative electron sink was observed among the studied okra accessions under drought-stress conditions. Drought-tolerant accessions indicated higher AES values. An increase in AES was reported as an indicator of drought stress [39]. Alternative electron sink is the second most important mechanism after photosynthesis used to remove electrons, which occurs at high rates in the leaves under drought stress conditions [40].

## 5. Conclusions

Drought is one of the most important factors affecting physiological traits and yield in crop plants, including okra. In the present study, it was observed that drought stress affected physiological processes such as reduced stomatal conductance, transpiration rate, net carbon dioxide assimilation, maximum quantum efficiency, effective quantum efficiency of *PSII* photochemistry, photochemical quenching and electron transport rate among the studied okra accessions. These physiological traits could be useful for drought-tolerance breeding in okra. Principal component analysis-based biplots allowed the identification of drought-tolerant accessions such as LS05, LS06, LS07 and LS08 based on high A, T, *Fm′*, *Fv′/Fm′* and ETR, and LS10, LS11, LS18 and LS23 based on high *AES*, *Ci*, *Ci/Ca*,*WUEi*, *WUEins*, *ɸPSII* and AES. The selected genotypes are high yielding (≥5 g/plant) under drought-stress conditions. These accessions are suitable candidates for parental genotypes for drought-tolerance breeding in okra to enhance water use efficiency under water-limited conditions.

## Figures and Tables

**Table 1:** Accession code, accession number, database, geographical origin, drought tolerance index and stem colour of the okra accessions evaluated in the study.

Accession Code	Accession Number	Database Name	Geographical Origin	DTI	Stem Colour
LS01	VI033775	ARC/South Africa	Malaysia	0.02	Red
LS02	VI033797	ARC/South Africa	Malaysia	1.16	Green
LS03	VI056457	ARC/South Africa	Yugoslavia	1.46	Red
LS04	VI039651	ARC/South Africa	Bangladesh	0.67	Green
LS05	VI046561	ARC/South Africa	Thailand	1.80	Red
LS06	VI047672	ARC/South Africa	Bangladesh	1.00	Green
LS07	VI050150	ARC/South Africa	Taiwan	0.13	Green
LS08	VI050957	ARC/South Africa	Zambia	0.04	Green
LS09	VI050960	ARC/South Africa	Zambia	0.31	Green
LS10	VI055110	ARC/South Africa	Malaysia	0.15	Red
LS11	VI055119	ARC/South Africa	Myanmar	0.73	Red
LS12	VI055219	ARC/South Africa	Malaysia	0.99	Red
LS13	VI055220	ARC/South Africa	Malaysia	4.67	Green
LS14	VI055421	ARC/South Africa	Viet Nam	1.02	Green
LS15	VI056069	ARC/South Africa	Cambodia	0.14	Red
LS16	VI056079	ARC/South Africa	Cambodia	3.15	Green
LS17	VI056081	ARC/South Africa	Cambodia	0.53	Red
LS18	VI056449	ARC/South Africa	United States of America	0.43	Red
LS19	VI060131	ARC/South Africa	Mali	0.00	Green
LS20	VI060313	ARC/South Africa	Tanzania	6.49	Green
LS21	VI060679	ARC/South Africa	India	0.61	Green
LS22	VI060803	ARC/South Africa	Turkey	8.64	Green
LS23	VI060817	ARC/South Africa	Brazil	0.45	Green
LS24	VI060822	ARC/South Africa	Nigeria	0.31	Green
LS25	VI060823	ARC/South Africa	Nigeria	0.00	Green
LS26	Clemson Spineless	ARC/South Africa	South Africa	0.23	Green

ARC = Agricultural Research Council, DTI = drought tolerance index.

**Table 2:** Analysis of variance indicating mean squares and significant tests of leaf gas exchange and chlorophyll fluorescence parameters of 26 okra genotypes evaluated under non-stress and drought-stress conditions averaged across two seasons.

**Source of Variation**	**d.f.**	**Leaf Gas Exchange Parameters**									
		***gs***	***T***	***A***	***Ci***	***A/C*** **_i_**	***C_i_/C_a_***	**WUE*_i_***	**WUE*_ins_***		
Replications	1	0.07 *	3.19 ^ns^	34.76 ^ns^	806,541 *	0.03 ^ns^	7.30 *	324,534 *	3675 ^ns^		
Incomplete Blocks	1	0.01 ^ns^	1.92 ^ns^	54.88 ^ns^	15,807 ^ns^	0.05 ^ns^	1.48 ^ns^	4185 ^ns^	685 ^ns^		
Genotype (G)	25	0.13 **	13.45 **	65.50 ^ns^	165,972 ^ns^	0.06 ^ns^	1.13 ^ns^	140,060 *	652,347 **		
Water Regime (WR)	1	0.38 **	75.62 **	448.16 **	1,830,530 *	0.04 ^ns^	20.94 **	3,444,897 **	20,180,480 **		
G × WR	25	0.01 ^ns^	14.46 **	30.41 *	100,917 ^ns^	0.06 ^ns^	1.03 ^ns^	140,099 *	644,150 **		
Residual	50	0.11	4.33	31.35	174,990	0.07	1	56,471	205,739		
**Source of Variation**	**d.f.**	**Chlorophyll Fluorescence Parameters**									**YPP**
		***F_O_*** **′**	***F_m_*** **′**	***F_v_′/F_m_′***	***ɸ*** ***PSII***	***qP***	***qN***	**ETR**	**ETR/A**	**AES**	
Replications	1	8892 ^ns^	32,989 ^ns^	0.22 *	0.08 ^ns^	0.11 ^ns^	0.25 ^ns^	3.72 ^ns^	586,500 ^ns^	2518 ^ns^	1186.7 *
Incomplete Blocks	1	8535 ^ns^	468,996 *	0.02 ^ns^	0.06 ^ns^	0.24 *	1.69 *	1.40 ^ns^	164,477 ^ns^	15,882 ^ns^	127.6 ^ns^
Genotype (G)	25	28,927 **	292,297 *	0.17 *	0.17 **	0.25 **	1.94 **	2.86 *	356,680 ^ns^	14,198 ^ns^	1023.2 *
Water Regime (WR)	1	844,279 **	20,220,415 **	0.66 **	1.35 **	1.20 **	8.68 **	4.16 ^ns^	101,913 **	424,290 **	6913.0 **
G × W	25	19,264 *	472,144 **	0.05 *	0.16 **	0.20 **	1.69 **	1.86 ^ns^	301,433 *	12,027 ^ns^	194.9 *
Residual	50	9080	115,681	0.08	0.03	0.14	0.38	1.2	156,859	11,175	183.9

d.f.: degree of freedom, *gs*: stomatal conductance, *T*: transpiration rate, *A*: net CO_2_ assimilation, *Ci*: intercellular CO_2_ concentration, *A*/*Ci*: CO_2_ assimilation rate/intercellular CO_2_ concentration, *Ci*/*Ca*: ratio of intercellular and atmospheric CO_2_, WUE*_i_*: intrinsic water use efficiency, WUE*_ins_*: instantaneous water use efficiency, *F*_0_′: minimum fluorescence, *Fm*′: maximum fluorescence, *Fv*′/*Fm*′: maximum quantum efficiency of photosystem II photochemistry, *ɸPSII*: the effective quantum efficiency of PSII photochemistry, *qP*: photochemical quenching, *qN*: non-photochemical quenching, ETR: electron transport rate, ETR/A: relative measure of electron transport to oxygen molecules, AES: alternative electron sinks, YPP: yield per plant, * and ** denote significance at 5 and 1% probability levels, respectively, ns: non-significant.

**Table 3:** Means of leaf gas exchange and chlorophyll fluorescence parameters of okra accessions under non-stressed conditions.

Genotype			Leaf Gas Exchange Parameters								Chlorophyll Fluorescence Parameters							YPP
	*gs*	T	A	*Ci*	A/*Ci*	*Ci*/*Ca*	WUE*i*	WUE*ins*	Fo′	Fm′	*Fv′/Fm′*	*ɸ* *PSII*	*qP*	*qN*	ETR	ETR/A	AES	
LS01	0.19	1.52	21.85	1.33	0.12	1.33	171.30	17.77	388.4	828.1	0.75	0.33	0.14	1.24	22,026	1129.9	56.59	7.02
LS02	0.3	1.06	27.91	0.74	0.15	0.74	162.40	26.39	302.9	787.7	0.41	0.19	0.19	2.23	30,068	1082.6	26.09	7.83
LS03	0.26	7.01	16.24	0.84	0.14	0.84	62.00	2.32	311.6	871.8	0.57	0.30	0.35	2.78	13,186	801.6	17.98	8.79
LS04	0.24	2.52	24.93	0.78	0.17	0.78	102.20	10.58	168.8	860	0.28	0.29	0.48	0.75	21,020	807.40	20.85	6.09
LS05	0.20	2.01	21.05	1.37	0.07	1.37	164.90	10.45	186.8	421.4	0.61	0.04	0.02	1.23	2815	137.10	33.70	7.33
LS06	0.23	6.56	26.95	0.68	0.15	0.68	195.90	4.18	179.7	252.3	0.37	0.37	0.25	0.74	8148	306.70	18.36	8.00
LS07	0.22	1.56	29.82	0.62	0.18	0.62	136.60	28.44	166.3	678	0.44	0.24	0.15	0.68	24,945	739.40	18.34	2.92
LS08	0.29	1.52	30.01	0.75	0.11	0.75	109.70	20.87	444.4	552.3	0.40	0.24	0.16	0.76	37,921	1226.80	26.13	2.63
LS09	0.39	4.02	21.02	1.65	0.14	1.65	55.10	11.61	227.4	822	0.64	0.40	0.14	1.24	23,498	1113.60	26.52	7.17
LS10	0.34	1.26	35.54	1.66	0.15	1.66	108	28.26	434	811.10	0.80	0.30	0.33	1.23	52,313	1464.40	18.63	6.88
LS11	0.27	1.01	28.85	0.78	0.11	0.78	107.40	28.44	207.10	139.6	0.53	0.37	0.14	2.31	23,806	776.30	14.05	9.23
LS12	0.29	5.52	23.9	0.59	0.16	0.59	80.40	5.90	263.6	769	0.35	0.29	0.83	1.22	24,860	1047.40	37.65	7.68
LS13	0.28	9.02	25.05	0.78	0.18	0.78	92.80	2.78	193.7	864.70	0.51	0.24	0.48	0.77	9184	378.90	15.30	6.13
LS14	0.19	2.02	29.08	1.90	0.17	1.90	173	14.42	391.3	845.40	0.44	0.28	0.13	2.76	26,106	852.30	15.70	8.56
LS15	0.50	7.56	29.91	0.66	0.13	0.66	61.30	4.39	370.7	472.90	0.93	0.37	0.26	1.76	37,168	1223.10	30.08	4.82
LS16	0.14	5.51	16.5	0.83	0.06	0.83	303.10	10.20	174.8	824	0.13	0.03	0.16	0.75	26,910	1553.60	24.12	0.01
LS17	0.53	2.51	22.08	0.71	0.12	0.71	41.40	12.74	289.8	794.50	0.74	0.31	0.36	1.24	21,657	961.70	33.09	6.00
LS18	0.22	6.41	27.08	0.69	0.18	0.69	142.80	4.43	355.9	903.20	0.45	0.43	0.15	1.77	18,416	690.20	22.91	6.10
LS19	0.32	8.02	28.84	0.61	0.18	0.61	88.70	3.60	260.5	692.70	0.36	0.40	0.12	1.69	26,298	918.80	18.44	11.55
LS20	0.14	1.22	21.77	1.00	0.12	1.00	163.30	57.12	79.6	909.80	0.59	0.00	0.10	2.80	6732	323.60	12.72	8.08
LS21	0.17	1.26	38.69	2.27	0.13	2.27	238.50	30.68	146.1	790.50	0.51	0.33	2.33	0.73	59,155	1528.80	61.12	9.58
LS22	0.24	1.11	22.39	0.98	0.10	0.98	98	55.81	321	229.10	0.48	0.17	0.33	1.78	17,721	763.20	12.77	11.44
LS23	0.16	7.51	21.38	1.73	0.18	1.73	168.70	3.25	124.2	155.40	0.49	0.27	0.2	0.82	20,206	939.50	32.53	8.00
LS24	0.27	9.26	27.77	1.34	0.10	1.34	102.60	3.07	229.5	500.20	0.50	0.25	0.10	0.80	35,469	1381.90	30.68	13.25
LS25	0.28	1.12	23.3	0.76	0.1	0.76	86.4	26.53	111.6	927.5	0.33	0.36	0.39	0.78	28,312	1215.7	59.69	7.04
LS26	0.33	3.57	23.68	0.69	0.11	0.69	73.1	7.91	200.6	495.2	0.57	0.09	0.13	1.25	28,203	1191.7	17.33	5.24
Mean	0.27	3.91	25.6	1.03	0.14	1.03	126.52	16.62	251.17	661.48	0.51	0.26	0.32	1.39	24,852	944.47	26.98	7.2
*p*-value	*	*	*	ns	ns	*	*	*	ns	**	*	**	**	**	ns	ns	ns	**
SED	0.09	2.23	5.5	201	0.44	0.51	52.13	19.36	122	191.1	0.16	0.08	0.23	0.43	13,977	459.9	15.5	4.35
LSD (5%)	0.18	4.59	11.35	415	0.09	1.05	107.4	29.87	251.3	393.6	0.33	0.16	0.47	0.89	28,786	947	32.06	5.55
CV (%)	32.2	55.97	25.52	51.74	32.79	39.54	41.2	48.47	48.58	28.89	31.66	30.25	71.11	31.04	56.24	48.69	47.7	33.78

*gs*: stomatal conductance (mmol m ^−2^ s^−1^), T: transpiration rate (mmol H_2_0 m ^−1^ s^−1^), A: net CO_2_ assimilation (µmol CO_2_ m^−1^ s^−1^), A/Ci: CO_2_ assimilation rate/intercellular CO_2_ concentration (µmol.mol ^−1^), Ci: intercellular CO_2_ concentration (µmol.mol ^−1^), Ci/Ca: ratio of intercellular and atmospheric CO_2_, WUEi: intrinsic water use efficiency ((µmol (CO_2_)m^−2^), WUEins: instantaneous water use efficiency (µmol.mol^−1^), F_0_′: minimum fluorescence, Fm′: maximum fluorescence, *Fv′/Fm′*: maximum quantum efficiency of photosystem II photochemistry (ratio), *ɸPSII*: the effective quantum efficiency of *PSII* photochemistry, *qP*: photochemical quenching, *qN*: non-photochemical quenching, ETR: electron transport rate (µmol e^−1^ m^−2^ s^−1)^, ETR/A: relative measure of electron transport to oxygen molecules (µmol e µmol^−1^ CO_2_), AES: alternative electron sinks, SED: standard deviation, YPP: yield per plant (g/plant), LSD: least significant difference, CV: coefficient of variation, * and ** denote significance at 5 and 1% probability levels, respectively, ns: non-significant.

**Table 4:** Means of leaf gas exchange and chlorophyll fluorescence parameters of okra accessions under drought-stressed conditions.

Genotype			Leaf Gas Exchange Parameters								Chlorophyll Fluorescence Parameters							YPP
	*gs*	T	A	*Ci*	A/*Ci*	*Ci*/*Ca*	WUE*i*	WUE*ins*	*Fo’*	*Fm′*	*Fv*′/*Fm*′	*ɸ* *PSII*	*qP*	*qN*	ETR	ETR/A	AES	
LS01	0.16	0.01	24.6	425.1	0.12	1.11	847.90	1881.90	442.20	1826	0.36	0.06	0.11	3.75	27,140	1111	263	3.92
LS02	0.31	1.01	29.03	316.1	0.23	0.80	193.30	1212.90	420.80	1733	0.40	0.05	0.11	3.72	19,975	682	116.60	2.58
LS03	0.16	0.01	11.11	216.9	0.14	3.55	72.60	1196.30	465.60	1775	0.33	0.05	0.13	0.72	19,196	1782	98.00	2.50
LS04	0.09	0.01	19.9	1064	0.07	1.97	225.80	2164.70	489.80	282	0.24	0.04	0.21	2.80	16,373	791	66.80	4.19
LS05	0.13	4.51	15.11	763.3	0.15	0.66	178.50	372.80	443.50	1890	0.41	0.00	0.05	1.78	10,688	683	562.80	6.17
LS06	0.12	2.51	15.27	920.7	0.05	2.86	312.20	696.50	54.80	1775	0.36	0.03	0.09	2.74	13,797	898	113.70	5.05
LS07	0.17	4.51	16.03	728.6	0.13	0.83	198.90	312.60	104.80	1746	0.49	0.04	0.06	0.71	18,534	1156	104.20	7.92
LS08	0.10	0.01	16.82	225.8	0.15	1.08	252.70	1354.60	509.10	1774	0.34	0.06	0.13	1.72	34,541	2044	86.00	6.58
LS09	0.29	0.01	24.22	881.6	0.05	2.79	98.60	1933.30	483.00	1598	0.38	0.10	0.11	0.66	39,986	1902	199.90	4.60
LS10	0.27	6.12	30.16	671.2	0.13	3.07	565.20	522.10	449.5	2867	0.37	0.04	0.03	3.7	16,452	551	263.50	14.00
LS11	0.03	0.01	20.65	1205.9	0.12	1.83	923.40	1986.30	506.8	1809	0.24	0.05	0.12	3.74	20,909	1007	70.40	6.76
LS12	0.21	0.01	22.17	221.40	0.14	0.57	155.90	1918.90	461.2	344	0.42	0.05	0.34	1.75	23,095	1057	111.50	2.85
LS13	0.02	2.01	25.32	1058	0.05	2.74	1438.8	734.60	505.7	640	0.32	0.05	0.10	2.67	23,537	871	112.80	2.00
LS14	0.17	0.51	24.54	290.40	0.10	1.25	311.4	761.80	519.20	963	0.34	0.06	0.16	0.69	24,880	1041	114.40	4.48
LS15	0.34	1.01	22.62	598.30	0.13	2.06	565.6	995.90	497.90	1805	0.35	0.06	0.16	1.74	29,353	1316	170.70	4.71
LS16	0.15	2.01	17.14	234.40	0.15	1.09	122.9	330.50	539	1533	0.37	0.02	0.09	1.32	8937	516	67.60	2.63
LS17	0.28	9.01	26.83	959.40	0.10	2.49	901.1	1048	429.90	806	0.38	0.09	0.19	2.71	41,445	1542	89.20	3.69
LS18	0.25	3.67	23.51	1167.80	0.09	1.71	696.1	601.70	478.40	1709	0.33	0.04	0.19	3.69	19,453	842	311.90	5.42
LS19	0.14	0.01	16.48	641.60	0.04	1.65	764.1	1392.50	373.80	1803	0.47	0.05	0.2	1.75	20,599	1228	91.50	0.50
LS20	0.02	3.51	24.42	909	0.05	2.36	1256.1	1039.60	449.40	1714	0.26	0.05	0.11	2.7	19,890	849	163.10	0.75
LS21	0.16	0.62	24.17	211.60	0.28	0.54	836.5	97.70	498.80	762	0.39	0.06	0.12	0.72	25,724	1072	82.20	4.17
LS22	0.10	0.01	21.33	718.40	0.08	1.85	476.4	2161	307	1826	0.33	0.03	0.05	0.8	8129	381	64.90	1.75
LS23	0.10	4.51	24.23	234.80	0.12	3.60	511.8	375.60	505.90	1651	0.23	0.05	0.09	0.67	10,832	485	154.50	5.88
LS24	0.26	3.66	24.96	863.40	0.10	3.23	297.5	257.80	518.20	1848	0.26	0.04	0.13	2.25	18,797	772	102.9	4.17
LS25	0.11	4.01	19.88	697.70	0.06	2.30	366.5	706.30	364.20	1774	0.30	0.05	0.00	0.78	21,796	1140	360.60	0.01
LS26	0.10	4.01	17.15	815.40	0.06	2.09	184.6	1379.50	397.10	1874	0.34	0.04	0.26	0.79	8071	466	80.00	4.00
Mean	0.16	2.20	21.45	644	0.11	1.93	490.55	1055.21	431.37	1543.35	0.35	0.05	0.13	1.96	20,851.1	1007.12	154.72	4.31
*p*-value	*	**	*	ns	ns	*	*	**	**	**	ns	*	ns	**	*	**	ns	*
SED	0.07	1.91	4.5	503	0.08	1.21	317.1	641.5	57.65	442.6	0.1	0.02	0.09	0.61	6837	304.6	148.1	2.25
LSD (5%)	0.14	3.92	9.3	1036	0.18	2.5	653.1	1321	118.7	911.6	0.21	0.04	0.19	1.23	14,081	627.3	305	4.21
CV (%)	42.44	46.4	20.98	76.77	74.92	62.98	64.65	71.47	13.36	28.68	28.96	39.83	69.85	30.87	32.79	30.25	95.71	25.76

*gs*: stomatal conductance (mmol m ^−2^ s^−1^), T: transpiration rate (mmol H_2_0 m ^−1^ s^−1^), A: net CO_2_ assimilation (µmol CO_2_ m^−1^ s^−1^), A/*Ci*: CO_2_ assimilation rate/intercellular CO_2_ concentration (µmol·mol ^−1^), *Ci*: intercellular CO_2_ concentration (µmol·mol ^−1^), *Ci*/*Ca*: ratio of intercellular and atmospheric CO_2_, WUE*i*: intrinsic water use efficiency (µmol (CO_2_)m^−2^), WUE*ins*: instantaneous water use efficiency (µmol·mol^−1^), *F*_0_′: minimum fluorescence, *Fm*′: maximum fluorescence, *Fv′/Fm′*: maximum quantum efficiency of photosystem II photochemistry (ratio), *ɸPSII*: the effective quantum efficiency of *PSII* photochemistry, *qP*: photochemical quenching, *qN*: non-photochemical quenching, ETR: electron transport rate (µmol e^−1^ m^−2^ s^−1)^, ETR/A: relative measure of electron transport to oxygen molecules (µmol e µmol^−1^ CO_2_), AES: alternative electron sinks, YPP: yield per plant (g/plant), SED: standard deviation, LSD: least significant difference, CV: coefficient of variation, * and ** denote significance at 5 and 1% probability levels, respectively, ns: non-significant.

**Table 5:** Correlation coefficients for gas exchange and chlorophyll fluorescence parameters under non-stressed (bottom diagonal) and drought-stressed (top diagonal) conditions.

Traits	*gs*	T	A	*C_i_*	A/*C_i_*	*C_i_*/*C_a_*	WUE*_i_*	WUE*_ins_*	*F_O_′*	*F_m_′*	*F_v_′/F_m_′*	*ɸ* *PSII*	*qP*	*qN*	ETR	ETR/A	AES	YPP
*gs*	1.00	0.17 ^ns^	0.57 *	−0.16 ^ns^	0.31 ^ns^	−0.18 ^ns^	−0.33 ^ns^	−0.13 ^ns^	0.19 ^ns^	0.23 ^ns^	0.47 *	0.54 **	0.14 ^ns^	0.11 ^ns^	0.45 *	0.21 ^ns^	0.18 ^ns^	0.25 ^ns^
T	0.13 ^ns^	1.00	0.23 ^ns^	0.30 ^ns^	−0.12 ^ns^	0.30 ^ns^	0.14 ^ns^	−0.55 **	−0.22 ^ns^	0.28 ^ns^	0.27 ^ns^	−0.07 ^ns^	−0.21 ^ns^	0.11 ^ns^	−0.16 ^ns^	0.24 ^ns^	0.45 *	0.31 ^ns^
A	0.14 ^ns^	−0.19 ^ns^	1.00	0.12 ^ns^	0.15 ^ns^	0.09 ^ns^	0.48 *	−0.34 ^ns^	0.46 ^ns^	−0.18 ^ns^	0.37 ^ns^	0.42 *	−0.03 ^ns^	0.43 *	0.45 *	−0.26 ^ns^	0.02 ^ns^	0.18 ^ns^
*Ci*	−0.31 ^ns^	−0.29 ^ns^	0.30 ^ns^	1.00	−0.61 **	0.31 ^ns^	0.38 ^ns^	0.26 ^ns^	−0.26 ^ns^	0.03 ^ns^	0.16 ^ns^	−0.03 ^ns^	−0.03 ^ns^	0.43 *	0.04 ^ns^	0.14 ^ns^	0.14 ^ns^	0.66 **
A/*Ci*	0.04 ^ns^	0.29 ^ns^	0.35 ^ns^	−0.02 ^ns^	1.00	−0.57 **	−0.28 ^ns^	−0.29 ^ns^	0.23 ^ns^	−0.16 ^ns^	0.03 ^ns^	−0.06 ^ns^	−0.07 ^ns^	0.01 ^ns^	0.15 ^ns^	0.01 ^ns^	0.03 ^ns^	0.24 ^ns^
*Ci*/*Ca*	−0.31 ^ns^	−0.29 ^ns^	0.38 ^ns^	1.00 **	−0.02 ^ns^	1.00	0.13 ^ns^	0.07 ^ns^	0.22 ^ns^	0.36 ^ns^	0.28 ^ns^	−0.19 ^ns^	−0.19 ^ns^	0.01 ^ns^	0.03 ^ns^	0.03 ^ns^	−0.19 ^ns^	0.67 **
WUE*i*	−0.75 **	−0.14 ^ns^	0.16 ^ns^	0.45 ^ns^	−0.20 ^ns^	0.35 ^ns^	1.00	−0.17 ^ns^	0.24 ^ns^	−0.12 ^ns^	0.33 ^ns^	0.22 ^ns^	−0.07 ^ns^	0.43 *	0.23 ^ns^	0.68 **	−0.04 ^ns^	0.48 *
WUE*ins*	−0.24 ^ns^	−0.74 **	0.16 ^ns^	0.13 ^ns^	−0.35 ^ns^	0.13 ^ns^	0.14 ^ns^	1.00	0.15 ^ns^	−0.29 ^ns^	0.03 ^ns^	0.30 ^ns^	0.39 *	0.16 ^ns^	0.29 ^ns^	0.23 ^ns^	0.27 ^ns^	−0.23 ^ns^
*Fo′*	0.36 ^ns^	−0.06 ^ns^	0.39 ^ns^	0.01 ^ns^	0.12 ^ns^	0.01 ^ns^	−0.35 ^ns^	−0.21 ^ns^	1.00	−0.22 ^ns^	0.14 ^ns^	0.24 ^ns^	0.24 ^ns^	0.18 ^ns^	0.24 ^ns^	0.18 ^ns^	0.03 ^ns^	0.83 **
*Fm*′	0.01 ^ns^	−0.12 ^ns^	−0.15 ^ns^	0.02 ^ns^	0.26 ^ns^	0.02 ^ns^	−0.01 ^ns^	−0.08 ^ns^	0.13 ^ns^	1.00	0.33 ^ns^	−0.27 ^ns^	0.55 **	0.13 ^ns^	−0.27 ^ns^	−0.12 ^ns^	0.25 ^ns^	0.40 *
*Fv′/Fm′*	0.21 ^ns^	−0.17 ^ns^	0.193 ^ns^	−0.12 ^ns^	0.23 ^ns^	−0.12 ^ns^	0.03 ^ns^	−0.18 ^ns^	00.21 ^ns^	0.27 ^ns^	1.00	0.46 *	00.08 ^ns^	0.197 ^ns^	0.53 **	0.19 ^ns^	0.28 ^ns^	0.36 ^ns^
*ɸ* *PSII*	0.38 ^ns^	0.36 ^ns^	0.42 ^ns^	0.02 ^ns^	0.51 **	0.02 ^ns^	−0.36 ^ns^	−0.44 ^ns^	0.30 ^ns^	0.04 ^ns^	0.05 ^ns^	1.00	0.23 ^ns^	0.16 ^ns^	0.87 **	0.67 **	0.29 ^ns^	−0.09 ^ns^
*qP*	−0.12 ^ns^	−0.24 ^ns^	0.55 *	0.48 *	0.08 ^ns^	0.48 *	0.23 ^ns^	0.13 ^ns^	−0.19 ^ns^	0.29 ^ns^	0.13 ^ns^	0.29 ^ns^	1.00	0.15 ^ns^	0.28 ^ns^	0.13 ^ns^	0.48 *	−0.21 ^ns^
*qN*	−0.01 ^ns^	−0.25 ^ns^	−0.13 ^ns^	−0.27 ^ns^	0.17 ^ns^	−0.27 ^ns^	−0.14 ^ns^	0.18 ^ns^	0.35 ^ns^	0.10 ^ns^	−0.12 ^ns^	−0.17 ^ns^	−0.25 ^ns^	1.00	0.08 ^ns^	−0.17 ^ns^	0.13 ^ns^	0.25 ^ns^
ETR	0.22 ^ns^	−0.19 ^ns^	0.71 *	0.38 ^ns^	−0.25 ^ns^	0.48 *	0.06 ^ns^	0.11 ^ns^	0.31 ^ns^	0.12 ^ns^	0.15 ^ns^	0.37 ^ns^	0.52 **	−0.24 ^ns^	1.00	0.82 **	0.07 ^ns^	−0.03 ^ns^
ETR/A	0.22 ^ns^	−0.16 ^ns^	0.38 ^ns^	0.25 ^ns^	−0.22 ^ns^	0.25 ^ns^	0.16 ^ns^	−0.15 ^ns^	0.37 ^ns^	0.23 ^ns^	0.27 ^ns^	0.27 ^ns^	0.31 ^ns^	−0.37 ^ns^	0.86 **	1.00	0.29 ^ns^	−0.60 **
AES	−0.03 ^ns^	−0.20 ^ns^	0.10 ^ns^	0.33 ^ns^	−0.33 ^ns^	0.33 ^ns^	0.27 ^ns^	−0.14 ^ns^	−0.10 ^ns^	0.21 ^ns^	0.12 ^ns^	0.38 ^ns^	0.52 **	−0.45 *	0.37 ^ns^	0.49 *	1.00	0.19 ^ns^
YPP	−0.29 ^ns^	−0.26 ^ns^	−0.69 **	0.45*	00.32 ^ns^	0.45*	0.37 ^ns^	0.35 ^ns^	0.35 ^ns^	−0.35 ^ns^	−0.04 ^ns^	−0.29 ^ns^	−0.23 ^ns^	0.22 ^ns^	0.16 ^ns^	0.11 ^ns^	0.16 ^ns^	1.00

*gs*: stomatal conductance, T: transpiration rate, A: net CO_2_ assimilation, A/*Ci*: CO_2_ assimilation rate/intercellular CO_2_ concentration, *Ci*: intercellular CO_2_ concentration, *Ci*/*Ca*: ratio of intercellular and atmospheric CO_2_, WUE*i*: intrinsic water use efficiency, WUE*ins*: instantaneous water use efficiency, F_0_′: minimum fluorescence, *Fm*′: maximum fluorescence, *Fv′/Fm′*: maximum quantum efficiency of photosystem II photochemistry (ratio), *ɸPSII*: the effective quantum efficiency of *PSII* photochemistry, *qP*: photochemical quenching, *qN*: non-photochemical quenching, ETR: electron transport rate, ETR/A: relative measure of electron transport to oxygen molecules, AES: alternative electron sinks, YPP: pod yield per plant. * and ** denote significance at 5 and 1% probability levels, respectively, ns: non-significant.

## Data Availability

Not applicable.

## References

[1] Abd El-Fattah B.E.S., Haridy A.G., Abbas H.S. (2020). Response to planting date, stress tolerance and genetic diversity analysis among okra (*Abelmoschus esculentus* (L.) Moench.) varieties. Genet. Resour. Crop Evol..

[2] Barzegar T., Moradi P., Nikbakht J., Ghahremani Z. (2016). Physiological response of Okra cv. Kano to foliar application of putrescine and humic acid under water deficit stress. Int. J. Hortic. Sci. Technol..

[3] Mkhabela S.S., Shimelis H., Gerrano A.S., Mashilo J. (2021). Phenotypic and genotypic divergence in okra [*Abelmoschus esculentus* (L.) Moench] and implications for drought tolerance breeding: A review. S. Afr. J. Botany.

[4] Gemede H.F., Ratta N., Haki G.D., Beyene A.Z.W.F. (2015). Nutritional Quality and Health Benefits of Okra (*Abelmoschus esculentus*): A Review. Glob. J. Med. Res. K Interdiscip..

[5] Petropoulos S., Fernandes A., Barros L., Ferreira I.C.F.R. (2018). Chemical composition, nutritional value and antioxidant properties of Mediterranean okra genotypes in relation to harvest stage. Food Chem..

[6] Romdhane M.H., Chadoura H., Barros L., Dias M.I., Correa R.C.G., Morales P., Gudad-Mulero M., Flamini G., Majdoub H., Ferreira I.C.F.R. (2020). Chemical composition, nutritional value, and biological evaluation of Tunisian okra pods (*Abelmoschus esculentus* L. Moench). Molecules.

[7] Mihretu Y., Wayessa G., Adugna D. (2014). Variability and association of quantitative characters among okra (*Abelmoschus esculentus* L.) Moench) Collection in South-Western Ethiopia. J. Biol. Sci..

[8] FAO (2020). Food and Agriculture Organization of the United Nations.

[9] Ayub Q., Khan S.M., Mehmood A., Haq N.U., Ali S., Ahmad T., Ayub M.U., Hassan M., Hayat U., Shoukat M.F. (2020). Enhancment of physuological and biochmical attributes of okra by application of salicylic acid under drought stress. J. Hortic. Sci. Technol..

[10] Iqbal S., Parveen N., Bahadur S., Ahmad T., Shauaib M., Nizamani M., Urooj Z., Zubab S. (2020). Paclobutrazol mediated changes in growth and physio-biochemical traits of okra (*Abelmoschus esculentus* L.) grown under drought stress. Gene Rep..

[11] Chaturvedi A.K., Surendran U., Gopinath G., Chandran K.M., Anjali N.K., Fasil M.C.T. (2019). Elucidation of stage specific physiological sensitivity of okra to drought stress through leaf gas exchange, spectral indices, growth and yield parameters. Agric. Water Manag..

[12] Duzyaman E. (2005). Phenotypic diversity within a collection of distinct okra (Abelmoschus esculentus) cultivars derived from Turkish land races. Genet. Resour. Crop Evol..

[13] Pravisya P., Jayaram K.M. (2015). Priming of *Abelmoschus esculentus* (L.) Moench (okra) seeds with liquid phosphobacterium: An approach to mitigate drought stress. Trop. Plant Res..

[14] dos Santos Farias D.B., da Silva P.S.O., Lucas A.A.T., de Freitas M.I., de Jesus Santos T., Fontes P.T.N., de Oliveira Júnior L.F.G. (2019). Physiological and productive parameters of the okra under irrigation levels. Sci. Hortic..

[15] Alake C.O. (2020). Genetic variability and diversity in okra landraces using agro-morphological traits and seed elemental minerals. Int. J. Veg. Sci..

[16] Munir M., Amjad M., Ziaf K., Ahmad A. (2016). Improving okra productivity by mitigating drought through foliar application of salicylic acid. Pak. J. Agric. Sci..

[17] Adejumo S.A., Ezeh O.S., Mur L.A.J. (2019). Okra growth and drought tolerance when exposed to water regimes at different growth stages. Int. J. Veg. Sci..

[18] Shi D., Wang J., Bai Y., Liu Y. (2020). Transcriptome sequencing of okra (*Abelmoschus esculentus* L. Moench) uncovers differently expressed genes responding to drought stress. J. Plant Biochem. Biotechnol..

[19] Mkhabela S.S., Shimelis H., Gerrano A.S., Mashilo J., Shayanowako A. (2022). Characterization of okra (*Abelmoschus esculentus* L.) accessions with variable drought tolerance throught simple sequence repeat markers and phenotypic traits. Diversity.

[20] Bindinger F.R., Mahalakshmi Y., Talukdar B.S., Alagarswamy G., IRRI (1982). Improvement of drought resistance in pearl millet. Drought Resistance in Crops with Emphasis on Rice.

[21] Kitao M., Lei T.T., Koike T., Tobita H., Maruyama Y. (2003). Higher electron transport rate oberved at low intercellular CO_2_ concentration in long term drought-acclimaed leaves of Japanese mountain birch (*Betula ermanii*). Physiol. Plant..

[22] Webster R.J., Driever S.M., Kromdijik J., McGrath J., Leakey A.D., Siebke K., Demetriades-Shah T., Bonnage S., Peloe T., Lawson T. (2016). High C_3_ photosynthetic capacity and high intrisic water use efficiency underlies the high productivity of the bioenergy grass Arundo donax. Sci. Rep..

[23] Medrano H., Tomas M., Martorell S., Flexas J., Hernandez E., Rossello J., Pou A., Escalona J.M., Bota J. (2015). From leaf to whole-plant water use efficiency (WUE) in complex canopies: Limitations of leaf WUE as a selection target. Crop J..

[24] Zlatev Z. (2009). Drought-indiced changes in chlorophyll flourescence of young wheat plants. Biotechnol. Biotechnol. Equip..

[25] Evans J.R. (2009). Potential errors in electron transport rates calculated from chlorophyll fluorescence as revelaed by a multilayer leaf model. Plant Cell Physiol..

[26] Ort D.R., Baker N.R. (2002). A photoprotective role of for O_2_ as an alternative electron sink in photosynthesis?. Curr. Opin. Plant Biol..

[27] Ahmed Z.G., El-Sayed M.A. (2021). Influence of drought stress on physiological traits of crossed okra varieties. Jordan J. Biol. Sci..

[28] Xu Z.Z., Jiang Y.L., Zhou G.S. (2015). Response and adaptation of photosynthesis, respiration and antioxidant systems to elevated CO_2_ with environmental stress in plants. Front. Plant Sci..

[29] Zwicke M., Picon-Cochard C., Morvan-Bertrand A., Prud’homme M.P., Volaire F. (2015). What functional strategies drive drought survival and recovery of perennial species from upland grassland?. Ann. Bot..

[30] Mandizvo T., Odindo A.O., Mashilo J., Magwaza L.S. (2022). Drought tolerance assessment of citron watermelon (*Citrullus lanatus var citroides* (L.H Bailey) Mansf.ex Greb.) accessions based on morphological and physiological traits. Plant Physiol. Biochem..

[31] Shu S., Yuan L.Y., Guo S.R., Sun J., Yuan Y.H. (2013). Effects of exogenous spermine on chlorophyll fluorescence, antioxidant system and ultrastructure of chloroplasts in *Cucumis sativus* L. under salt stress. Plant Physiol. Biochem..

[32] Rahbarian R., Khavari-Nejad R., Ganjeali A., Bagheri A., Najafi F. (2011). Drought stress effects on photosyntheisi, chlorophyll fluorescence and water relations in tolerant and susceptible chickpean (*Cicer Arietinum,* L.) genotypes. Acta Biol. Crac. Ser. Bot..

[33] Paknejad F., Nasri M., Reza H.M.T., Zahedi H., Jami M.A. (2007). Effects of drought stress on chlorophyll flourescence parameters, chlorophyll content and grain yield of wheat cultivars. J. Biol. Sci..

[34] Zivcak M., Brestic M., Balatova Z., Drevenakova P., Olsovska K., Kalaji H.M., Yang X., Allakhverdiev S.I. (2013). Photosynthetic electron transport and specific photoprotective responses in wheat leaves under drought stress. Photosynth. Res..

[35] Nar H., Saglam A., Terzi R. (2009). Leaf rolling and photosystem II in *Ctenanthe setosa* exposed to drought stress. Photosynthetica.

[36] Johnson G.N. (2011). Physiology of PSI cyclic electron transport in higher plants. Biochim. Et Biophys. Acta.

[37] Ashraf M., Arfan M., Shahbaz M., Ahmad A., Jamil A. (2002). Gas exchange characteristics and water relations in some elite okra culivars under water deficit. Photosynthetica.

[38] Guo Y.Y., Yu H.Y., Kong D.D., Yan F., Zhang Y.J. (2016). Effects of drought stress on growth and chlorophyll fluorescence of *Lycium ruthenicum* Murr. Seedlings. Photosynthetica.

[39] Yi X., Zhang Y., Yao H., Zhnga X., Luo H., Gou L., Zhang W. (2014). Alternative electron sinks are crucial for conferring photoprotection in field-grown cotton under water deficit during flowering and boll setting stages. Funct. Plant Biol..

[40] Foyer C.H., Bloom A.J., Queval G., Noctor G. (2009). Photorespiratory metabolism: Genes, mutants, energetics, and redox signaling. Annu. Rev. Plant Biol..

